# Flexible and Stretchable Microneedle Patches with Integrated Rigid Stainless Steel Microneedles for Transdermal Biointerfacing

**DOI:** 10.1371/journal.pone.0166330

**Published:** 2016-12-09

**Authors:** Mina Rajabi, Niclas Roxhed, Reza Zandi Shafagh, Tommy Haraldson, Andreas Christin Fischer, Wouter van der Wijngaart, Göran Stemme, Frank Niklaus

**Affiliations:** 1 KTH Royal Institute of Technology, School of Electrical Engineering, Department of Micro and Nanosystems, Stockholm, Sweden; 2 Karlsruhe Institute of Technology (KIT), Institute of Nanotechnology, Karlsruhe, Germany; Institute of Materials Research and Engineering, SINGAPORE

## Abstract

This paper demonstrates flexible and stretchable microneedle patches that combine soft and flexible base substrates with hard and sharp stainless steel microneedles. An elastomeric polymer base enables conformal contact between the microneedle patch and the complex topography and texture of the underlying skin, while robust and sharp stainless steel microneedles reliably pierce the outer layers of the skin. The flexible microneedle patches have been realized by magnetically assembling short stainless steel microneedles into a flexible polymer supporting base. In our experimental investigation, the microneedle patches were applied to human skin and an excellent adaptation of the patch to the wrinkles and deformations of the skin was verified, while at the same time the microneedles reliably penetrate the surface of the skin. The unobtrusive flexible and stretchable microneedle patches have great potential for transdermal biointerfacing in a variety of emerging applications such as transdermal drug delivery, bioelectric treatments and wearable bio-electronics for health and fitness monitoring.

## Introduction

Recent advances in flexible and stretchable electronics show great potential for wearable bioelectronics and epidermal sensor systems that are in contact with the surface of the human skin, e.g. for measuring physiological signals in diagnostics, and health and fitness monitoring applications [1,2]. Flexible and stretchable skin-like substrates are able to conformally attach to the surface of the skin and deform along with the skin without detaching, thereby offering enhanced wearer comfort. Previously, flexible and stretchable epidermal patches have been developed and demonstrated to monitor physiological parameters such as electrophysiological signals, skin temperature, skin hydration and sweat and movement disorders [1–13]. In contrast to epidermal patches, microneedles pierce and penetrate into the upper layers of the skin. Microneedles typically are 100–1000 μm long, which is sufficiently short to avoid touching nerve endings in the lower layers of the skin, thereby enabling minimally invasive and painless insertion of the microneedles into the skin [14–16]. Consequently, transdermal interfacing with microneedle arrays has interesting applications, including transdermal delivery of drugs, extraction of physiological signals with high quality, accessing body-fluids for real-time monitoring of biochemical parameters (e.g. glucose, pH level and other diagnostic markers), skin treatments in cosmetics and bioelectric treatments (electroceuticals) [14–21]. While flexible and stretchable patches with polymer microneedles have been reported [22–27], to the best of our knowledge flexible and stretchable patches in combination with sharp and stiff stainless steel microneedle arrays have not been demonstrated to date. A large number of studies on microneedle geometries, materials and fabrication methods have been reported [18]. Microneedles have been made of materials such as silicon, metals, polymers, glass and ceramics and a variety of fabrication methods have been used for fabricating microneedle arrays, including semiconductor micromachining, laser cutting and micromolding [17–25]. Practically, all microneedle arrays today are realized as monolithic components in which both the microneedles and the supporting base substrate are made of the same material [14–20]. This necessarily requires tradeoffs between the desired structural properties of the microneedles and the base substrate. While microneedles need to be mechanically robust and rigid to avoid fracturing, and sufficiently sharp to enable easy penetration into the skin, the base substrate should be flexible to conformally adapt to the curvature and deformations of the human skin to reduce the risk for detachment of the microneedle patch from the skin and improve wearer comfort. Monolithic polymer microneedle arrays can potentially provide some level of flexibility of the supporting base substrate. However, in this approach the use of a soft polymer can compromise the mechanical strength and sharpness of the microneedle tips, which in turn may make it difficult to insert the microneedles into the skin in a reliable way [28]. While two-layered polymer technology [29] can in principle be used to realize microneedle patches with needles made of a stiff polymer in combination with a soft polymer substrate, to the best of our knowledge such a device has not yet been demonstrated.

Stainless steel needles are widely used in the medical industry, are low cost and readily commercially available, and the employed assembly technology is scalable and potentially allows for low-cost fabrication [30]. In the present paper, we introduce and demonstrate microneedle patches that combine a soft and flexible polymer base substrate with mechanically strong and sharp stainless steel microneedles. The soft elastomeric supporting base enables conformal contact between the microneedle patch and the curved surface of the skin, while the sharp stainless steel microneedles penetrate the skin. A magnetic assembly technology is employed for integrating the stainless steel microneedles into the flexible base substrate. This magnetic assembly technology was previously demonstrated for filling of through silicon via holes with nickel wires, in which an array of 30×30 via holes was filled within 140 sec [30] and several via hole arrays were assembled in parallel using multiple magnets[31], indicating the suitability of this approach for scalable manufacturing. The proposed flexible microneedle patches for transdermal biointerfacing could facilitate various emerging applications in the area of wearable electronics, including transdermal drug delivery, real-time health and fitness monitoring, diagnostics, and bioelectric therapies.

## Materials and Methods

### Substrate preparation

**Fig 1:**
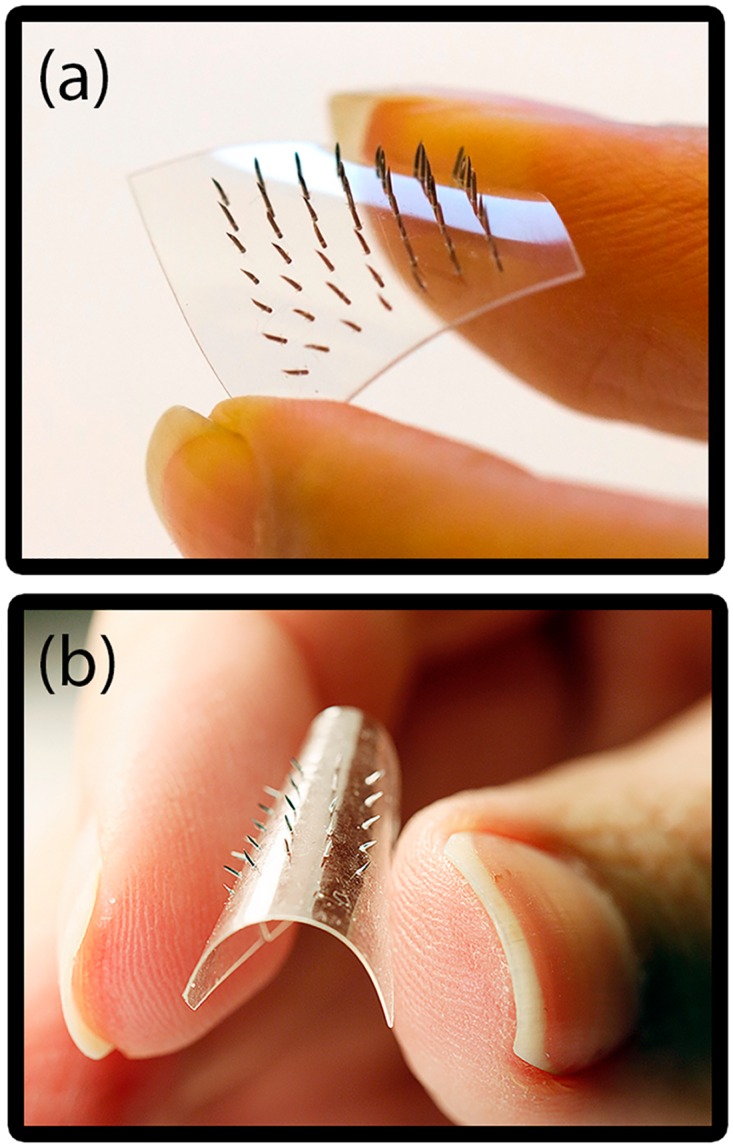
Photographs of the fabricated microneedle patches. (a) Microneedle patch type A (higher Young’s modulus and lower flexibility). (b) Microneedle patch type B (lower Young’s modulus and higher flexibility).

In this work we have realized and evaluated microneedle patches with two different elasticities of the base substrate (patch type A and B), which are shown in Fig 1a and 1b respectively. In microneedle patch type A, the base substrate consists of a polystyrene film (Shrinkles^®^, Panduro Hobby, Sweden) with a thickness of 250 μm, which is a commonly available flexible polymer film that can be used to realize microneedle patches with a comparably robust and flexible base substrate. An array of 7×7 holes was drilled in the polystyrene film using a CNC milling machine. The holes have a diameter of 220 μm and are placed with a pitch of 2 mm. The polymer film is then cut into an area of 18×18 mm^2^ to further proceed with the assembly process. In microneedle patch type B, the base substrate consists of a molded thiol-ene-epoxy-based thermoset film (OSTEMER Flex, Mercene Labs AB, Sweden). The OSTEMER was chosen because it is flexible, stretchable and because it allows scalable microstructuring and needle fixation by covalent bonding. To obtain improved bond strength between the stainless steel microneedles and the base substrate, a two-step polymerization process for the base substrate was used [32]. Therefore, the liquid resin of the OSTE flex polymer was injected into a milled aluminum mold and then exposed to collimated UV-light (14 mWcm^-2^) for 100 s to start the first polymerization step, thereby partly crosslinking the polymer. The mold contains an array of 5×5 pillars, where each pillar defines a hole in the replicated base substrate (S1 Fig). The pillars have a diameter of approximately 210 μm and are placed with a pitch of 2 mm. The top surface of the mold was covered with a laminated release liner (ScotchPak 9775 Release liner, 3M, USA) on a poly methyl methacrylate (PMMA) plate to aid the demolding process. The demolded, partially cured polymer base substrate is 100 μm thick and has a size of 20×20 mm^2^. Patches with the thickness of 200 μm were realized for evaluating mechanical properties and detachment forces and 100 μm for the insertion tests.

### Microneedles

The needles used for patch type A are 31 gauge stainless steel bevel needles (Unifine^®^ Pentips^®^, Owen Mumford Ltd, UK) with an initial length of 18 mm and a nominal outer diameter of 250 μm. The needles were shortened to a length of 2 mm by aligning the needles on the beveled side and cutting them using a diamond blade dicing saw (ZH05, Disco Corporation, Japan). For patch type B, 32 gauge stainless steel bevel needles (Novo Nordisk AB, Denmark), with an initial length of 16 mm and a nominal diameter of 220 μm were used. The needles were shortened to a length of 1.1 mm, by aligning the needles on the beveled side and cutting them using a laser microfabrication workstation (Laser μFAB, equipped with a Spectra-Physics Spirit 1040-4/-SHG femtosecond laser, Newport, USA). The main reason to use different gauges of the needles in patch type A and B was that we selected the needles with the smallest possible diameter available at the time of the experiments. The lengths of the needles were adjusted in line with the length of the bevel of the needles and the thickness of the base substrate.

### Magnetic assembly of flexible microneedle patch

**Fig 2:**
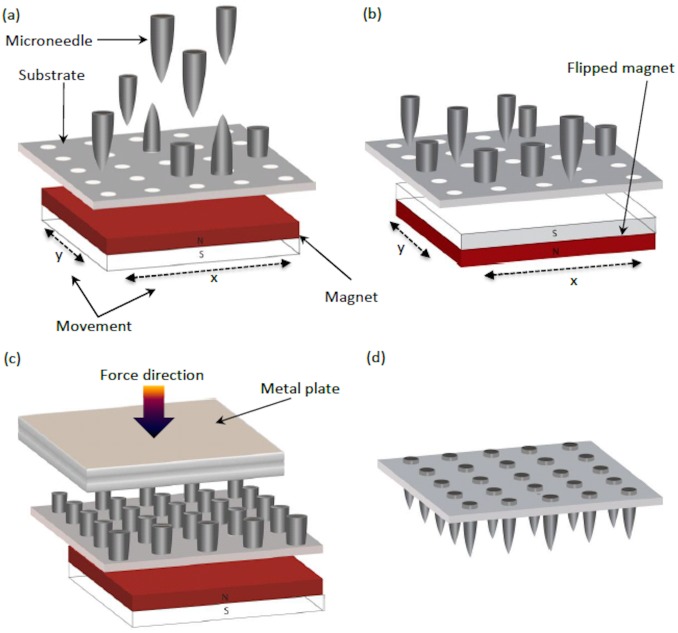
Illustration of the magnetic assembly process for the microneedles patches. (a) A permanent magnet is placed about 5 mm below the flexible base substrate and an excess amount of microneedles is dispensed onto the substrate. The ferromagnetic microneedles align along the field lines of the magnetic field perpendicular to the substrate surface. As the magnet is moved laterally, the microneedles follow the magnets and the needles with the bevel facing to the substrate surface get trapped inside the holes. (b) By flipping the magnets upside down, the microneedles that are not trapped in holes rotate and turn the beveled end towards the substrate surface. (c) A metal plate is used to push the microneedles inside the holes. (d) 3D schematic of a fully assembled microneedle patch.

The ferromagnetic stainless steel microneedles were magnetically assembled into the holes of the base substrate as schematically illustrated in Fig 2. In the assembly process, the base substrate was pressed between two identical aluminum plates with a thickness of 1 mm and a cavity of 15×15×1 mm^3^ drilled in the center of the plates. In this way, all the substrate edges were fixed in between the plates and the microneedles can protrude out from the cavity. Two permanent magnets with dimensions of 15×15×3 mm^3^ (Q-15-15-03-N, Supermagnete, Germany) were placed 5 mm below the substrate on a holder that could move freely in the x-y plane. Two magnets were paired to provide a magnetic field sufficiently strong to position the needles vertically inside the holes. An excess amount of microneedles (about 30% more microneedles than holes) was placed onto the base substrate. The magnetic field aligned the microneedles perpendicularly to the substrate surface. Part of the microneedles had their beveled end facing down to the substrate and part had their beveled end facing upwards (Fig 2a). By moving the magnets in the x-y plane, the microneedles moved on the substrate surface until the ones with the beveled end facing to the substrate surface were trapped and partially inserted into the holes in the substrate. After being exposed to the magnetic field, the ferromagnetic microneedles were magnetized. Hence, by flipping the permanent magnet upside down, the polarity of the magnetic field changed and the microneedles that were not trapped in the holes of the base substrate rotated, thereby exposing their beveled end towards the substrate surface (Fig 2b). Continued movement of the magnets in the x-y plane resulted in filling of all the holes with microneedles. Finally, a metal plate was used to apply a force onto the end of the microneedles, thereby pushing the microneedles into the holes (Fig 2c). As the holes in the base substrate have a diameter that is slightly smaller than the diameter of the microneedles, a press-fit is established between the sidewalls of the holes and the microneedles. After the assembly, the excess needles were directed outside the substrate by moving the magnet accordingly. In the finished microneedle patch the needle tips extend outside of the base substrate by approximately 1300 μm for patch type A, (Fig 2d). In patch type B, the needle tips extend outside of the base substrate by approximately 950 μm. After assembly, the microneedle patch type B was placed in an oven at 70°C for 2 h to complete the polymerization of the substrate. As a result, the remaining unreacted functional groups in the polymer base substrate completely polymerize and form covalent bonds, via the epoxy groups, to the surface of the stainless steel microneedles in the holes, thereby providing a firm attachment between the microneedles and the base substrate.

## Results and Discussion

### Mechanical properties of the substrates

**Fig 3:**
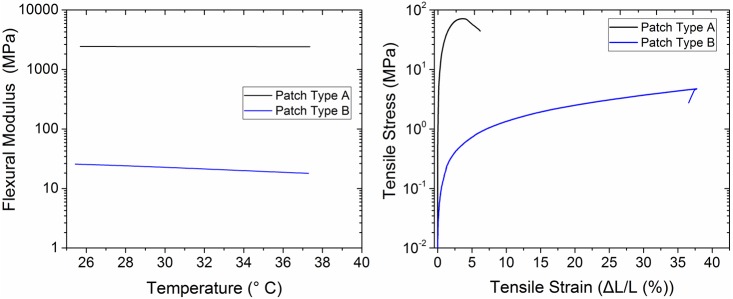
Mechanical properties of the base substrate type A and B. a) Representative flexural modulus as a function of temperature measured using the DMTA Q800. b) Representative tensile stress-strain curves measured using the Instron Microtester 5848.

To characterize the flexibility and the stretchability of the base substrates, the flexural modulus, Young’s modulus and maximum elongation at the point of breakage have been evaluated. Fig 3a shows the representative flexural modulus as a function of temperature for the base substrates of microneedle patch type A and B, measured using a dynamic mechanical thermal analysis instrument (DMTA Q800, TA Instruments, USA). The flexural modulus provides an inverse measure of the flexibility of a material. Flexural modulus of 2432 MPa and 29±4 MPa at 27°C were obtained for the base substrate type A and B, respectively. The flexural modulus of the base substrate of patch type B is almost two orders of magnitude lower than the flexural modulus of the base substrate of patch type A. The Young’s modulus of the base substrate type A and B were measured using an Instron Microtester (Instron 5944, USA) in tensile configuration. Prior to the experiments, all samples were conditioned in 50% relative humidity and 23°C for at least 48 h. The measurements were performed at a strain rate of 10%/min until the breaking point of the sample. Fig 3b shows representative stress-strain curves of base substrates type A and B. Table 1 shows the Young’s modulus, % elongations and tensile stresses at breakage extracted from the stress-strain curves. Base substrate type A features a limited elongation of only 3.5±0.5% and a high Young’s modulus of 1540±36 MPa. Base substrate type B features a very high elongation of 35±1% and a Young’s modulus of 15.5±0.5 MPa.

**Table 1:** Young’s modulus, % elongation and tensile stress at breakage, extracted from the tensile strain-strain curves of base substrates type A and B.

Base Substrate Type	Young’s Modulus (MPa)		Elongation at Breakage (%)		Tensile Stress at Breakage (MPa)	
	Mean	Standard Deviation	Mean	Standard Deviation	Mean	Standard Deviation
**A**	1540	36	3.5	0.5	69	1.8
**B**	15.5	0.5	35	1	5.4	0.4

### Evaluation of the skin insertion

**Fig 4:**
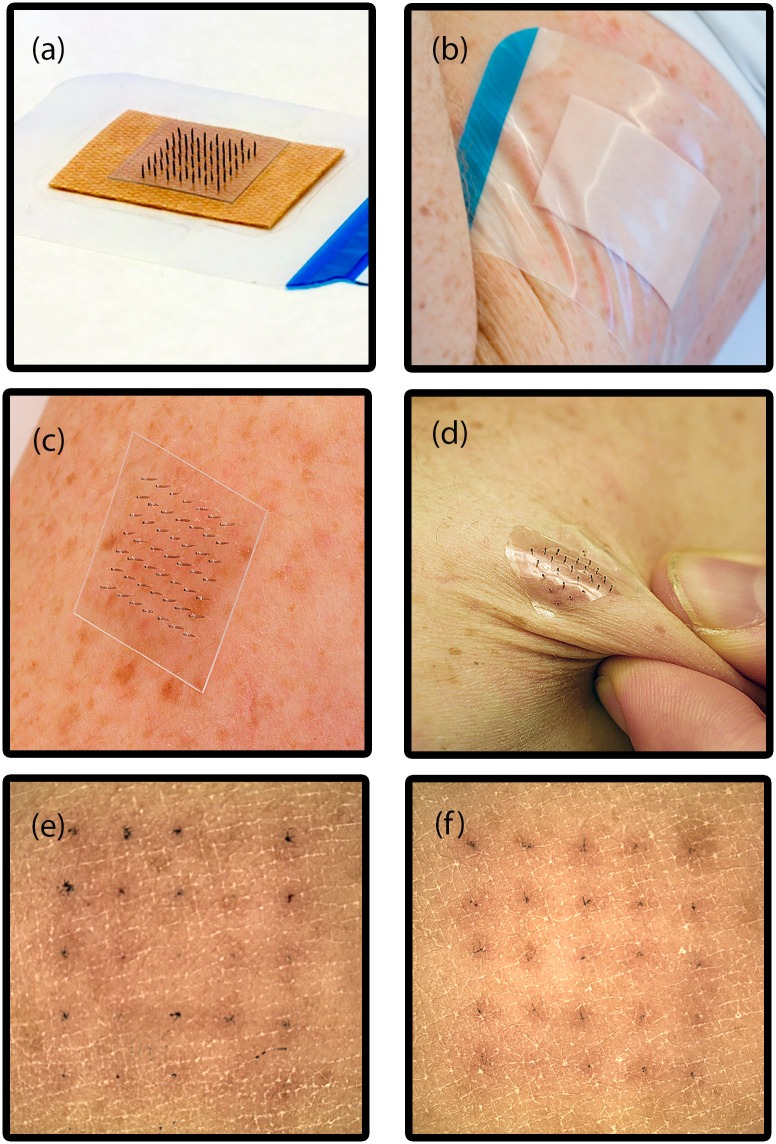
(a) Microneedle patch (type A) that is laminated to a plaster and (b) the same patch applied to the skin of a human arm. Photographs of microneedle patch type A (c) and type B (d) attached to the skin of the arm of the human subject. Microscope images of the insertion sites after removal of the microneedle patch type A (e) and type B (f) and after wiping off the excess dye. Each stained spot corresponds to the site of a microneedle penetrating the skin.

To evaluate the functionality and characteristics of the two different microneedle patches, they were applied onto the upper arm of a volunteer human subject (37 years old male) by manually pressing the patch with the thumb to the surface of the skin. All experiments have been approved by the Ethical Review Board in Stockholm (Dnr: 2015/867-31/1) and a written informed consent to participate in the study was provided by the volunteer. In the experiments, the microneedle patches were covered with a regular plaster (Aqua stop, Salvemed, Sweden) after application to the subject (Fig 4a and 4b). Fig 4c and 4d show applied microneedle patches without a plaster. It was observed that microneedle patch type A (Fig 4c) followed the body curvature but did not follow the wrinkles induced on the skin. In contrast, microneedle patch type B followed nicely the deformations of the soft skin without detaching (Fig 4d). The volunteer subject reported no perception of pain during wearing of the microneedle patches for a continuous period of 30 min with normal body activity. To verify insertion of the microneedles into the skin, a second set of experiments was performed in which the microneedle tips were immersed in a vessel containing liquid black food dye (Dr. Oekter, Germany) in a way that the bevel is covered with dye prior to application of the flexible patches to the upper arm of the human subject (37 years old male). For both microneedle patch type A and B, microscope images of the insertion sites were taken after the microneedle patch had been carefully removed and the excess dye had been wiped off (Fig 4e and 4f for patch type A and B, respectively). The remaining dye spots under the skin indicate that all microneedles had successfully pierced the skin. After the removal of the microneedle patches from the skin, in all cases the microneedles remained attached to the base substrates. For each of the microneedle patches type A and B, two insertions were successfully performed.

### Measurement of force required to detach the microneedles from the base substrate

**Fig 5:**
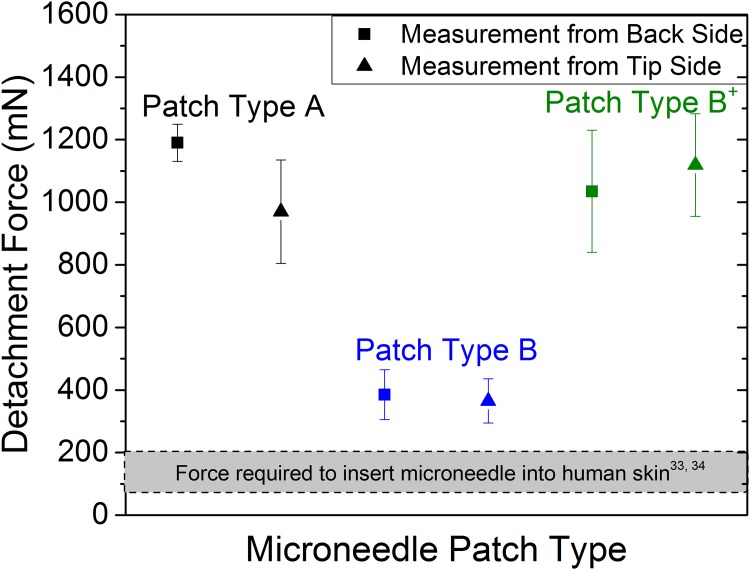
Needle detachment forces measured for patch type A and B when pulling from both, the needle tip and needle backside. The gray area represents the required force reported in literature to insert a microneedle into the human skin.

A figure of merit to assess the viability of the microneedle patches and penetration reliability of the microneedles into the skin is the force needed to detach the microneedles from the microneedle patch i.e. the minimum force required to displace the needles from the base substrate. Reports in literature indicate that forces in the range of 80–200 mN are required to insert microneedles similar to the ones used in our experiments into human skin [33,34]. The force needed to detach the needles from the base substrate is preferably considerably higher than the force required to insert the needle into the skin [35]. To assess the strength of the attachment between the microneedles and the base substrates, microneedles detachment forces were measured using a digital strain gauge (Lyman Electronic Trigger Pull Gauge, USA) and a bond tester (Dage 2400PC, Nordson DAGE, UK), (S2 Fig). Measurements of the force required to detach the needles from the base substrate were performed on patches that were used in the insertion experiments. Needle detachment forces for the used patch type A are in the range of 750–1200 mN while for the patch type B, the detachment forces are measured to have a relatively wide range of 45–1000 mN. Closer investigation of needles with very low detachment force revealed that the base substrate had microcracks around the microneedles, originating from the skin insertion experiments performed prior to the detachment force measurements. As noted above, all microneedles remained in the base substrate upon removing the patches from the skin. The lower detachment forces measured for patch type B compared to patch type A can be primarily attributed to the thinner base substrate of patch type B and to its much lower elastic modulus, which negatively impacts the press-fit between the base substrate and the microneedles. Additional detachment force measurements were performed on microneedle patches that have not been used in insertion experiments. For each microneedle patch type A and B, two different patches were used (four patches in total) and 15 needles were measured on each patch. For each patch 7 needles were pushed from the needle tip and another 8 needles were pushed from the needle backside. To evaluate the possibility to increase the attachment strength of the needles to the base substrate, in this experiment the thickness of the base substrate of microneedle patch type B was set to 200 μm. Fig 5 shows the detachment force of the different patch types measured when pulling from both the needle tip and the needle backside. The gray area in the Fig 5 indicates the approximate force required for inserting a microneedle into the human skin [33,34]. The detachment force for patch type A and type B were measured to be 1190±60 mN and 385±80 mN from the needle backside and 970±165 mN and 365±70 mN from the needle tip, respectively. Although the detachment force for the microneedle patch type B was lower compared to type A, they are still considerably higher than the skin insertion force. To further increase the bond strength between the microneedles and the base substrate in patch type B, an improved patch type B^+^, was synthesized, in which an additional 20 mul of 10% diluted OSTEMER was coated on the back side of the patch after microneedle assembly. The needle detachment force for patch type B^+^ improved by 170% as compared to patch type B, and was measured to be 1034±196 mN when pulling the needles from their backside and 1119±164 mN when pulling the needles from the tip. An alternative possibility to further increase the attachment strength between the microneedles and the base substrate may be the use of microneedles with small and short enlargements of the needle diameters at the needle ends that can be embedded in the base substrate, thereby realizing a form fit between the microneedles and the base substrate.

## Conclusion

In summary, we have demonstrated the feasibility of a flexible microneedle patch composed of a soft and flexible base substrate in combination with stiff and sharp stainless steel microneedles. The elastomeric polymer base enables conformal contact between the microneedle patch and the complex topography of the underlying skin, and the sharp microneedles reliably pierce into the skin. While our flexible microneedle patches display full functional integrity, we found that the attachment between the microneedles and the substrate needs special attention, especially if thin and highly flexible base substrate materials are used. Improved attachment strength between stainless steel microneedles and a thin elastomeric base substrate could for example be achieved by using microneedles with widened microneedle ends and embedding these widened ends with the base substrate material. The type of unobtrusive flexible and stretchable microneedle patch described in this work could play an important role in emerging applications such as in transdermal drug delivery, real-time health and fitness monitoring, diagnostics, and bioelectric therapies.

## Supporting Information

S1 File Details of the mold insert for the base substrate of microneedle patch type B. (DOCX)

S2 File Details of setup for measuring needle detachment force. (DOCX)

S1 Fig Illustrations of the mold insert. (TIF)

S2 Fig Illustration of the bond test equipment (Dage PC2400, Nordson DAGE, UK) and the setup for measuring the detachment force between the base substrate and the microneedles. To fasten the microneedle patch, a holder with 500 μm diameter holes and a pitch of 2 mm (identical to the pitch of the microneedle array) was fabricated. A stripe with one row of microneedles was cut from the microneedle patch and placed on a holder in a way that each microneedle was located inside a hole of the holder. Using a microscope, the stage of the bond test equipment was carefully adjusted in a way that the probe was perpendicular to the microneedle. The inset illustrates a magnified side view of the needle-probe arrangment. During a measurement, the motorized probe moves laterally towards the microneedle and records the force required to detach the microneedle from the base substrate.
(TIF)
