# Supplementary material for: Flexible and Stretchable Microneedle Patches with Integrated Rigid Stainless Steel Microneedles for Transdermal Biointerfacing
Source: PLoS One. 2016 Dec 9;11(12):e0166330. doi: 10.1371/journal.pone.0166330 (PMC5147815; doi:10.1371/journal.pone.0166330)
Supplement: S1 File — (DOCX) [file pone.0166330.s001.docx]

**Supporting information**

**S1 File. Details of the mold insert for the base substrate of microneedle patch type B**

A mold insert was made by milling a 5 mm thick Al (alloy 6082-T) with a high-precision CNC milling machine. The mold consists of a 20 × 20 mm^2^ wide and 100 µm thick cavity, accommodating an array of 5×5 pillars, with a pillar diameter of 210 µm, a pillar length of 100 µm and a pillar separation distance of 2 mm. The cavity also contains an injection port and a ventilation port, each with a diameter of 4 mm. During molding of the base substrate, a PMMA plate was covered with a release liner (ScotchPak 9775 Release liner, 3M, USA). The PMMA plate with release liner facing surface of the mold was placed on top of the mold and was clamped with paper clips. The liquid OSTE Flex resin was injected into the mold cavity through the injection port using a standard syringe.

**S1 Fig. Illustrations of the mold insert.**
