# Supplementary material for: Flexible and Stretchable Microneedle Patches with Integrated Rigid Stainless Steel Microneedles for Transdermal Biointerfacing
Source: PLoS One. 2016 Dec 9;11(12):e0166330. doi: 10.1371/journal.pone.0166330 (PMC5147815; doi:10.1371/journal.pone.0166330)
Supplement: S2 File — (DOCX) [file pone.0166330.s002.docx]

**S2 File. Details of setup for measuring needle detachment force**

**S2 Fig. Illustration of the bond test equipment (Dage PC2400, Nordson DAGE, UK) and the setup for measuring the detachment force between the base substrate and the microneedles. To fasten the microneedle patch, a holder with 500 µm diameter holes and a pitch of 2 mm (identical to the pitch of the microneedle array) was fabricated. A stripe with one row of microneedles was cut from the microneedle patch and placed on a holder in a way that each microneedle was located inside a hole of the holder. Using a microscope, the stage of the bond test equipment was carefully adjusted in a way that the probe was perpendicular to the microneedle. The inset illustrates a magnified side view of the needle-probe arrangment. During a measurement, the motorized probe moves laterally towards the microneedle and records the force required to detach the microneedle from the base substrate.**
