# Supplementary figures and images for: Flexible and Stretchable Microneedle Patches with Integrated Rigid Stainless Steel Microneedles for Transdermal Biointerfacing
Source: PLoS One. 2016 Dec 9;11(12):e0166330. doi: 10.1371/journal.pone.0166330 (PMC5147815; doi:10.1371/journal.pone.0166330)

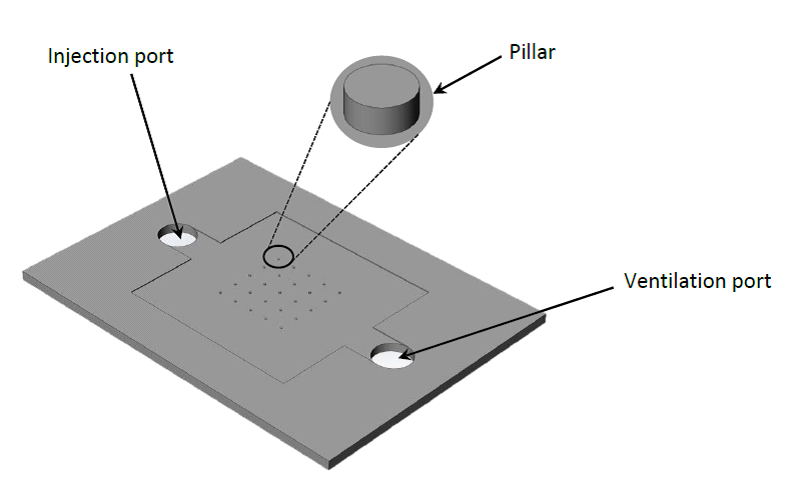

Supplement: S1 Fig — (TIF) [file pone.0166330.s003.tif]

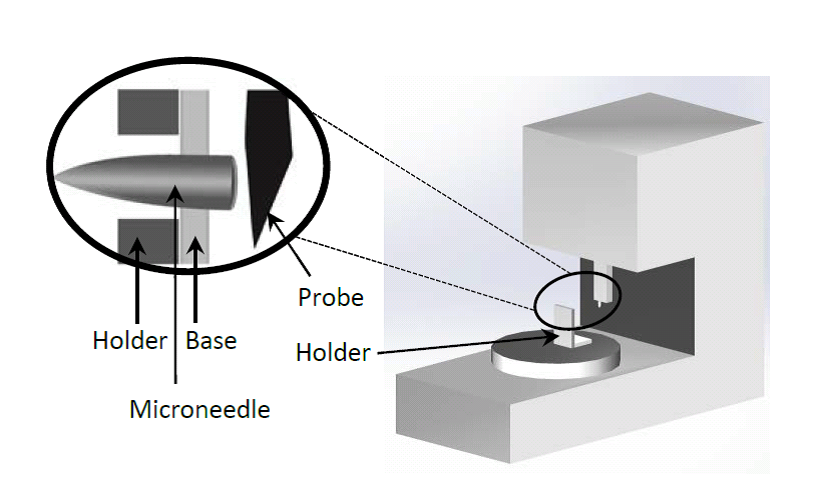

Supplement: S2 Fig — To fasten the microneedle patch, a holder with 500 μm diameter holes and a pitch of 2 mm (identical to the pitch of the microneedle array) was fabricated. A stripe with one row of microneedles was cut from the microneedle patch and placed on a holder in a way that each microneedle was located inside a hole of the holder. Using a microscope, the stage of the bond test equipment was carefully adjusted in a way that the probe was perpendicular to the microneedle. The inset illustrates a magnified side view of the needle-probe arrangment. During a measurement, the motorized probe moves laterally towards the microneedle and records the force required to detach the microneedle from the base substrate. (TIF) [file pone.0166330.s004.tif]
